# E-learning vs conventional teaching among students during CoVid-19 pandemic in India

**DOI:** 10.6026/973206300181005

**Published:** 2022-10-31

**Authors:** Chaitra MC, Akhil J, Lathasri Bhimagani, Navya YS, Lavanya SJ, Meghana Kunchapu

**Affiliations:** 1 Sri Devaraj Urs Medical College, Constituent of Sri Devaraj Urs Academy of Higher Education and Research, Kolar, Karnataka, India

**Keywords:** E-learning, Allied Health Sciences, traditional, conventional teaching, students, satisfaction

## Abstract

Internet technology is considered as an important medium for many aspects, including academic learning. E-learning ("e" for electronic)has received much attention in the recent years globally, E-learning pioneer explains "e" as exciting, energetic, enthusiastic, emotional, extended, and educational. Second year students of Allied Health Sciences of all courses studying in Sri Devaraj Urs Academy of Higher Education & Research, Tamaka, Kolar, India were included in the study. A set of semi-structured questionnaire was distributed to the students based on the effectiveness of learning through e-classes and their understanding with the satisfaction level.A total of 124 students of mean age group was between 20 ± 1.45 years. 62% were females and 38% were male in this study. The mean score and percentage of all the parameters assessed suggest that online learning was less effective when compared to the conventional classroom teaching. It implies that e-learning is not as effective or superior teaching method for students in the learning context especially the practical aspect.

## Background:

Sudden Covid-19 pandemic created panic & anxiety among the population worldwide. It was the academics which got affected the most due to pandemic. [1] Nowadays, technology has obviously made our lives easier. Internet technology is considered as an important medium for many aspects, including academic learning. E-learning ("e" for electronic) has received much attention in the recent years globally, with an estimated 5-7millon students now enrolling in at least one online course each year [2]. E-learning pioneer explains "e" as exciting, energetic, enthusiastic, emotional, extended, and educational. It is a store house of education, information, communication, training, knowledge, and performance. [1] In a review, students have preferred web tutorials compared to traditional lectures-for easy accessibility, good quality of image, and repeat practice possibility. Web-based learning due to continuous development and updating has become an important tool in evidence-based medicine. The important limitations to online learning are student isolation and technical problems, they miss classroom interaction. To overcome these drawbacks, integrating classroom problem-solving sessions with online web-based education are necessary. In the past decade, there is an increase in e-learning in higher education offering some form of distance education. [3] During lockdown, it was a challenging time for professional courses. This was overcome through online classes. However, due to resource limitations in a developing country like India, this approach created a major challenge. To combat the crisis, colleges adopted various innovative techniques, used different software/applications such as zoom, Microsoft, Google classroom, and Google docs to take the online classes. These online classes/e-learning were started with an intention not only to complete the course but also to remain in continuous touch with the students, to increase the confidence and faith of the students in their faculty during Covid-19 pandemic. There are fewer studies among Allied Health Sciences students. This study has been taken to know the difference in outcome between traditional and online learning.

## Methodology:

This study is across sectional study conducted from January 2022 to April 2022. Second year students of Allied Health Sciences of all courses as the first year subjects are same, studying in Sri Devaraj Urs Academy of Higher Education & Research, Tamaka, Kolar were included in the study.

## Sample size:

Sample size (124) for the present study was estimated based on the study by Nimrat Kaur [1]. Considering power of 80% with 95% confidence to detect a mean difference of 2 in mean scores was completed.

## Objectives:

[1] To determine the effectiveness of e-learning VS conventional teaching among the under graduate students

[2] To determine the satisfactory level of students with regard to both online and offline classes

## Inclusion criteria:

Students who attended e-learning & conventional learning classes

## Exclusion criteria:

Students less than 75% attendance

Institutional Ethical clearance was obtained. Informed consent from all the participants was taken before the start of study. Before the start of the questionnaire, the students were asked about their state of mind during lockdown. A set of semi-structured questionnaire was distributed to the students based on the effectiveness of learning through e-classes and their understanding with the satisfaction level.This set of questionnaire was self-designed based on 5-point Likert scale. This was pre-tested on 20 students for standardization. All students voluntarily participated in the survey.

## Results:

A total of 124 students of various courses of Allied Health Sciences participated in the study. The mean age group was between 20 ± 1.45 years. 62% of the students were female and 38% were male in this study. Regarding the state of the mind during pandemic, it was found that 35.7 % students were anxious, 30% no change, 23.8% were relaxed, 9.5% were depressed, and 1% were euphoric (Figure 1 - see PDF). In this study in 11 out of 13 parameters, students rated the online - classes to be some what less effective. The parameters are about offering convenience, interaction level, individual learning needs, balancing of practical and theoretical knowledge, doubt sessions, the weekly learning time after class during the period of physical class room or online class, the learning effectiveness and the courage to speak during physical class room and online class room, evaluation is easy and fair, understanding through recorded classes, effective communication and grooming of professional career. In building skills & knowledge as well as effective communication, majority of students found that the online learning and traditional method as equally effective. The satisfactory level with regard to both online and conventional classes about class material, the balance of practical and theoretical knowledge students & availability of e-resources was found to be neutral. With regard to professional development and availability of assistance they were dissatisfied. Though a small percentage of students (10-15%) are strongly dissatisfied, it cannot be ignored as even 1 student has to be given equal importance in education.

## Discussion:

Covid-19 outbreak created a panic, distress among students. Online classes were started throughout schools and colleges keeping in mind, the student's perception. The mean score and percentage of all the parameters assessed suggest that online learning was less effective when compared to the conventional classroom teaching. It only implies that e-learning is not as effective or superior teaching method for students in the learning context especially the practical aspect. The main difference between types of classes is the mode of interaction between instructor and students as well as among the students. The results of this study indicate that although student performance is independent of mode of instruction, certain courses are more challenging to students who persist in virtual environment than in classrooms. According to another study, the effectiveness could also be influenced by characteristics of the students such as gender [5], attitude [6], satisfaction [7], and level of engagement [8] which is in accordance with our study, in which there is also gender variation and that can be contributing factor. A study by Bettinger et al. [9] shows that to some extent, online learning might not compete with aspects of other learning, such as interactive knowledge building between teacher and students. Such limitations could create opportunities for students to obtain self-learning methods through information technology. The results of this study were also similar to our survey which showed that the level of interaction of students with the faculty is less as compared to classroom teaching. According to Nalini et al. [10] highly personalized content for learning can be improved by web-based learning. The student's online expertise is possibly increased by diversity of skills and knowledge. No one method of teaching would be as effective as a combination/blended teaching, which is also being supported by Dodiya et al. [4].

## Conclusions:

Digitalization / Smart classroom has brought about a revolution in the field of Health sciences with the innovation of trinity of e-learning, e-teaching, and e research constituting the superstructure of e-education. This facilitates adaptive and collaborative learning by the learners and the teachers. Blended learning would lead to more development of professional skills and grooming of professional career.
